# Is CMV DNAemia an early marker of CMV colitis in patients with active ulcerative colitis?

**DOI:** 10.1128/spectrum.01159-24

**Published:** 2024-10-14

**Authors:** Laura Melotti, Matteo Rinaldi, Marco Salice, Nikolas K. Dussias, Nicholas Vanigli, Carlo Calabrese, Eleonora Scaioli, Liliana Gabrielli, Tiziana Lazzarotto, Francesca Rosini, Pierluigi Viale, Paolo Gionchetti, Maddalena Giannella, Fernando Rizzello

**Affiliations:** 1 Department of Medical and Surgical Sciences, Alma Mater Studiorum University of Bologna, Bologna, Italy; 2 Dept of Medical and Surgical Sciences, IBD Unit- IRCCS Azienda Ospedaliero-Universitaria- Policlinico Sant'Orsola-Malpighi, Bologna, Italy; 3 Infectious Disease Unit, Department for Integrated Risk Management, IRCCS Azienda Ospedaliero-Universitaria di Bologna, Bologna, Italy; 4 Microbiology Unit, IRCCS Azienda Ospedaliero-Universitaria di Bologna, Bologna, Italy; 5 Pathology Unit, IRCCS Azienda Ospedaliero-Universitaria di Bologna, Bologna, Italy; University of Siena, Siena, Italy

**Keywords:** CMV colitis, inflammatory bowel diseases, colectomy

## Abstract

Cytomegalovirus (CMV) colitis is a serious concern worsening the prognosis of patients with ulcerative colitis (UC). We aimed to assess risk factors and prognostic impact of CMV colitis in patients with moderate-to-severe UC flare. We conducted a retrospective, observational, single-center study. Consecutive adult patients hospitalized for moderate-to-severe UC from January 2020 to June 2023 were included. The primary endpoint was a diagnosis of CMV-colitis according to immunohistochemistry on tissue biopsies. The secondary endpoint was the need for colectomy within 30 days. Overall, 135 patients were included. CMV colitis was diagnosed in *n* = 37 (27.4%): *n* = 19 (51.4%) endoscopically, the remaining on surgical specimens. Of them, *n* = 23 (62.2%) had positive CMV-DNAemia with a median value of 1,008 cp/mL (interquartile range 318–2,980). Differences between the two groups (CMV colitis vs non-CMV) included age (60 vs 41 years, *P* = 0.004), Charlson Comorbidity Index (1 vs 0, *P* = 0.003), steroid refractoriness (86.5% vs 62.2%, *P* = 0.007), and positive CMV-DNAemia (62.2% vs 10.1%, *P* < 0.001). At multivariable analysis, steroid-refractory disease, Charlson Comorbidity Index, and CMV-DNAemia were associated with CMV colitis. Overall, *n* = 54 (39.7%) patients underwent colectomy, and this was significantly more common in patients with CMV colitis vs non-CMV group (54.1% vs 34.4%, *P* = 0.049). Kaplan-Meier showed that antiviral therapy seems to have a relevant impact on colectomy (*P* < 0.001). CMV-DNA blood detection is independently associated with CMV-positive refractory UC. Since CMV colitis may increase the risk of colectomy and antiviral treatment seems to reduce such risk, prospective studies are needed to confirm the role of CMV-DNA blood detection to early diagnose CMV colitis.

**IMPORTANCE:**

Cytomegalovirus (CMV) colonic reactivation worsens the prognosis of patients with active ulcerative colitis. Blood CMV-DNA reactivation is strongly associated with CMV colitis. Prompt diagnosis and treatment of CMV colitis can avoid surgery in most cases.

## INTRODUCTION

The role of cytomegalovirus (CMV) infection in patients affected by ulcerative colitis (UC) is still to be fully understood. CMV concomitant colitis in active UC is associated with steroid refractoriness and with poorer outcomes such as colectomy, need of rescue therapy, and increase of disease flares according to some retrospective studies (1–3). These evidences led the European Crohn’s and Colitis Organization guidelines to state that patients with refractory inflammatory bowel disease (IBD) should be tested for CMV infection. However, there are still some uncertainties regarding the role of CMV in exacerbating the severity of UC and whether antiviral treatment has a prognostic impact (4).

Besides the doubts about its pathogenic role, CMV colitis is also quite hard to diagnose when concomitant active UC is present. Several tests are available including histology, serology, and PCR for CMV DNA in the blood or intestinal tissue. However, blood testing and standard histology are considered to have a low sensitivity. Immunohistochemistry (IHC), possibly combined with tissue PCR (tPCR), is the gold standard; notably, no cut-off level is currently available (5). It is pivotal to differentiate between non-clinically relevant CMV blood detection and CMV colitis since CMV invasion of the colonic mucosa has been associated with poorer outcomes (4). Additionally, the identification of a marker able to stratify patients at high risk for CMV colitis could be very useful to reduce the time to appropriate management of immunosuppressive and antiviral therapy (5). Indeed, the timing of the diagnosis is particularly relevant since uncontrolled UC may rapidly evolve, and results from histopathology are usually available only after the introduction of high doses of steroids or even after surgery. Therefore, there is a strong need for early markers able to stratify patients at the highest risk of concomitant CMV colitis.

Thus, we aimed to investigate the clinical and laboratory characteristics of adult patients hospitalized for UC flare, the rate and levels of blood CMV viral load according to the diagnosis of CMV colitis, as well as the impact of antiviral treatment on the need for 30-day colectomy.

## MATERIALS AND METHODS

### Study design and setting

This retrospective observational study was carried out from January 2020 until June 2023 at IRCCS S. Orsola Hospital, a tertiary-university hospital in Bologna, Italy. We included consecutive patients with moderate-to-severe UC flare requiring hospitalization that underwent a gastrointestinal endoscopic procedure. After hospital admission, all patients were tested according to local clinical practice, including blood exams and microbiological work-up, including stools cultures, *Clostridioides difficile* toxin test, blood cultures, and CMV and Ebstein Barr virus (EBV) blood viremia. The study was approved by the Ethic Committee of the promoting center (n° 677/2023/Oss/AOUBo). Informed consent was obtained contacting patients via email or phone call. In the case of deceased or unreachable patients, the informed consent was waived considering the observational nature of the study.

### Study definitions and endpoint variables

Primary endpoint: CMV colitis diagnosis established with IHC and histological examination of colonic biopsy tissues. Main exposure variable for primary endpoint: CMV DNAemia defined as detection of any CMV viral load on blood specimens. Secondary outcome: surgery within 30 days from hospitalization defined as the need for non-elective colectomy. Other exposure variables included age, sex, comorbidities according to the Charlson Comorbidity Index (CCI) (6) and previous UC-related treatments. Disease activity was defined as mild, moderate, and severe colitis; according to the Truelove and Witts score (7), blood tests and results of microbiological investigations, colonic biopsy result, and disease management at baseline were also recorded. Administration of anti-CMV drugs was collected. An assay of CMV-DNA was performed on EDTA-anticoagulated whole blood samples by using a commercial quantitative real-time PCR. DNA extraction was performed with the QIAsymphony SP instrument (Qiagen, Hilden, Germany), and the quantification of CMV-DNA was performed by using a CMV ELITe MGB kit (ELITech Group, Turin, Italy) on the ABI Prism 7500 real-time PCR System (PE Applied Biosystem, Foster City, CA, USA). The extraction and amplification procedures were performed as previously described (8). The analytical sensitivity of the assay is 10 copies of target DNA per amplification reaction. The lower limit of quantification of the assay is 300 copies/mL whole blood. Steroid-refractory disease was defined as active disease despite an adequate dose and duration of steroid treatment (prednisone, 0.75–1 mg/kg day orally for at least 2 weeks; methylprednisolone, 1 mg/kg day intravenously for 1 week), according to IGIBD Guidelines (9). Clinical remission was evaluated at 30 days from hospitalization and defined as no rectal bleeding and no urgency with the normalization of inflammatory markers (10).

### Statistical analysis

Categorical variables were reported as counts and percentages. Continuous variables were expressed as mean ± SD if normally distributed, or as median and interquartile range (IQR) if non-normally distributed. For the univariate analysis, categorical variables were compared using *χ*^2^ test or Fisher’s exact test as appropriate, whereas continuous variables were compared using Student’s *t* test or Mann–Whitney U test depending on whether normally distributed or not. To investigate risk factors for CMV colitis, a logistic regression model including relevant clinical variables and statistically significant variables (*P* < 0.05) identified at univariate analysis was performed. For CMV-DNAemia, the optimal cut-off was obtained by calculating the area under the receiver operating characteristic (ROC) curve for observed data. Furthermore, sensitivity, specificity, positive and negative predictive values, and accuracy in predicting CMV colitis were evaluated for the selected cut-off. A Kaplan-Meier analysis was performed to assess different survival outcomes between groups. All the analyses were carried out using SPSS statistical software.

## RESULTS

### Study population characteristics

Overall 135 patients hospitalized for moderate-to-severe UC flare were enrolled during the study period. The median age was 45 (30–60) years, and 54.8% of patients were male (see Table 1). The median body mass index (BMI) at hospital admission was 21 (18–24), with a median Charlson Comorbidity Index of 0 (0–1). Enrolled patients received a prior diagnosis of UC within a median of 5 (1–12) years from hospitalization, and almost half (58/135, 42.6%) of them had a previous hospitalization for moderate-to-severe UC flare. Pancolitis at UC diagnosis was more frequent (64/135, 47.1%), and the majority of patients were previously treated with oral mesalazine or steroids (121/135, 89% and 121/135, 89%, respectively). Among previous biological drugs, infliximab (57/135, 41.9%) followed by vedolizumab (43/135, 31.6%) was more frequently administered. The median number of biological therapies per patient was 2 (1–3). Up to two-thirds of patients have been treated with high-dose steroids (≥20 mg/day of prednisolone) for at least 2 weeks within 1 month from hospitalization. At hospital admission, 109/135 (82.0%) of patients had a severe Truelove and Witts score, while the median Mayo endoscopic score was 3 (2–3). Pancolitis was confirmed endoscopically in 64/135 (47.4%) patients during hospitalization. CMV-DNA was tested at hospital admission in all cases, being positive in 24.3% of patients, whereas EBV-DNAemia was negative/clinically not relevant in all tested patients. More than two-thirds of patients were managed with high-dose intravenous steroids (102/135, 75.0%). Clinical remission was obtained in 44.1% (60/135) of cases within 30 days from hospital admission. Overall, 54/135 (39.7%) patients underwent colectomy within 30 days from hospitalization. One patient (0.7%) died within 30 days after colectomy for surgical complications. Overall, concomitant CMV colitis was confirmed in 37/135 (27.4%) patients. Diagnosis performed with IHC was made on endoscopic samples in 51.4% of cases, and in the remaining 48.6% on surgical specimens (Table 2). Fifteen out of 37 (40.5%) patients were treated with ganciclovir for a median time of 21 (18–24) days. In 13 out of 15 (86.7%) cases, antiviral treatment was shifted to oral valganciclovir. In none of the treated patients, a CMV refractory disease was suspected, as follow-up blood CMV-DNA values decreased until negativization.

**TABLE 1:** Comparison between patients hospitalized for UC with and without CMV colitis^*a*^

	W/o CMV colitis (*N* = 98, 72.6%)	CMV colitis (*N* = 37, 27.4%)	Overall (*N* = 135)	*P*-value
Demographic data				
Age (years), median (IQR)	41 (28–56)	60 (36–66)	45 (30–60)	0.004
Sex, male	53 (54.1)	21 (56.8)	74 (54.8)	0.848
Baseline characteristics				
Active smoker	16 (16.2)	3 (8.1)	19 (14.0)	0.278
Previous smoke	31 (31.3)	16 (43.2)	47 (34.6)	0.226
BMI (median, IQR)	21 (18–24)	22 (19–25)	21 (18–24)	0.564
Charlson comorbidity index, median (IQR)	0 (0–1)	1 (0–2)	0 (0–1)	0.003
Previous hospitalization for UC	40 (40.4)	18 (48.6)	58 (42.6)	0.438
Previous infection (90 days)	9 (9.1)	8 (21.6)	17 (12.5)	0.077
Bacterial infection	6 (6.1)	6 (16.2)	12 (8.8)	0.087
Viral infection	3 (3.0)	3 (8.1)	6 (4.4)	0.344
Previous antibiotic exposure (90 days)	14 (14.3)	9 (24.3)	23 (17.0)	0.201
UC diagnosis to hospitalization (years; median, IQR)	5 (1–12)	4 (1–12)	5 (1–12)	0.483
UC localization at diagnosis				
Proctitis	8 (8.1)	2 (5.4)	10 (7.4)	0.728
Proctosigmoiditis	24 (24.2)	9 (24.3)	33 (24.3)	1.000
Left colitis	20 (20.2)	6 (16.2)	26 (19.1)	0.807
Pancolitis	45 (45.5)	19 (51.4)	64 (47.1)	0.567
Previous treatments				
Topic mesalazine	64 (35.4)	19 (51.4)	83 (61.0)	0.171
Oral mesalazine	87 (87.9)	34 (91.9)	121 (89.0)	0.759
Topic steroids	50 (50.5)	18 (48.6)	68 (50.0)	1.000
Oral steroids	88 (88.9)	33 (89.2)	121 (89.0)	1.000
Budesonide	11 (11.1)	5 (13.5)	16 (11.8)	0.766
Beclometasone	28 (28.3)	10 (27.0)	38 (27.9)	1.000
Budesonide + beclometasone	31 (31.3)	13 (35.1)	44 (32.4)	0.685
Methylprednisolone	34 (34.3)	17 (45.9)	51 (37.5)	0.236
Prednisone	42 (42.4)	14 (37.8)	56 (41.2)	0.698
Methylprednisone + prednisone	70 (70.7)	28 (75.7)	98 (72.1)	0.670
High-dose steroids (>10 mg/day) for at least 2 weeks within 1 month	63 (64.3)	26 (70.3)	89 (65.9)	0.549
Previous immunosuppressors				
Infliximab	42 (42.4)	15 (40.5)	57 (41.9)	1.000
Adalimumab	20 (20.2)	6 (16.2)	26 (19.1)	0.807
Vedolizumab	33 (33.3)	10 (27.0)	43 (31.6)	0.539
Ustekinumab	4 (4.0)	2 (5.4)	6 (4.4)	0.663
Azatioprine	26 (26.3)	10 (27.0)	36 (26.5)	1.000
Mercaptopurine	7 (7.1)	1 (2.7)	8 (5.9)	0.447
Tofacitinib	4 (4.0)	0 (0)	4 (2.9)	0.574
Number of prior IS (median, IQR)	2 (1–3)	2 (1–4)	2 (1–3)	0.969
Disease severity at hospitalization				
Steroid-refractory disease	61 (62.2)	32 (86.5)	93 (68.9)	0.007
Blood in stools				0.767
Not present	14 (14.1)	5 (13.5)	19 (14.0)	
<50%	19 (19.2)	5 (13.5)	24 (17.6)	
>50%	66 (66.7)	27 (73.0)	93 (68.4)	
Number evacuations/day	10 (5–12)	8 (6–15)	10 (5–12)	0.248
Temperature >37.8°C	77 (77.8)	26 (70.3)	103 (75.7)	0.376
Pulse rate >90 bpm	71 (71.7)	21 (56.8)	92 (67.6)	0.105
Truelove and Witts score				0.208
Mild	1 (1.0)	0 (0)	1 (0.8)	
Moderate	20 (20.6)	3 (8.3)	23 (17.3)	
Severe	76 (78.4)	33 (91.7)	109 (82.0)	
Endoscopic Mayo score	3 (2, 3)	3 (3)	3 (2, 3)	0.050
Disease extension at hospital admission				0.013
Proctitis	2 (2.2)	1 (2.7)	3 (2.4)	
Proctosigmoiditis	30 (33.3)	3 (8.1)	33 (26.0)	
Left colitis	16 (17.8)	11 (29.7)	27 (21.3)	
Pancolitis	42 (46.7)	22 (59.5)	64 (47.4)	
Blood tests				
WBC (median, /mmc; IQR)	10.1 (7.9–12.6)	10.2 (7.1–13.6)	10.2 (7.7–12.7)	0.992
Lymphocytes (median, /mmc; IQR)	1.5 (1.0–2.1)	1.5 (0.6–2.5)	1.5 (0.9–2.1)	0.642
PLT (median, /mmc; IQR)	345 (257–449)	314 (226–431)	340 (240–436)	0.372
Hb (median, g/dL; IQR)	12.1 (9.9–13.7)	12.1 (9.4–13.6)	12.1 (9.9–13.7)	0.701
ESR (median, mm; IQR)	26 (16–50)	26 (19–43)	26 (16–47)	0.988
Creatinine (median, mg/dL; IQR)	0.77 (0.63–0.94)	0.81 (0.67–0.98)	0.79 (0.64–0.95)	0.327
Albumin (median, g/dL; IQR)	3.6 (3.2–3.9)	3.3 (3.0–3.8)	3.5 (3.1–3.9)	0.153
Total bilirubin (median, mg/dL; IQR)	0.51 (0.38–0.68)	0.64 (0.46–0.90)	0.55 (0.41–0.72)	0.013
CRP (median, mg/dL; IQR)	2.5 (0.7–6.7)	3.2 (1.7–6.8)	2.8 (0.8–6.7)	0.393
Management at hospitalization				
Topic mesalazine	18 (18.2)	6 (16.2)	24 (17.6)	1.000
Oral mesalazine	56 (56.6)	27 (73.0)	83 (61.0)	0.113
Topic steroids	18 (18.2)	0 (0)	18 (13.2)	0.003
Oral steroids	73 (73.7)	29 (78.4)	102 (75.0)	0.661
Methylprednisone	28 (28.3)	19 (51.4)	47 (34.6)	0.015
Intravenous steroids (0.75–1 mg/kg/day)	70 (70.7)	32 (86.5)	102 (75.0)	0.075
Infliximab	27 (27.3)	7 (18.9)	34 (25.0)	0.379
Vedolizumab	9 (9.1)	4 (10.8)	13 (9.6)	0.750
Ustekinumab	6 (6.1)	0 (0)	6 (4.4)	0.189
Positive blood CMV-DNAemia	10 (10.1)	23 (62.2)	33 (24.3)	<0.001
Blood CMV-DNA (cp/mL; median, IQR)	300 (300–467)	1008 (318–2980)	492 (300–1909)	0.013
Clinical assessment at 30 days from diagnosis				0.450
Remission	47 (47.5)	13 (35.1)	60 (44.1)	
Lack of response	51 (51.5)	24 (64.9)	75 (55.1)	
Reactivation	1 (1.0)	0 (0)	1 (0.7)	
Needing of rescue therapy	31 (31.3)	12 (32.4)	43 (31.6)	1.000
Outcomes				
CMV infection relapse at 30 days	0 (0)	1 (2.7)	1 (0.7)	0.272
Needing of surgery within 30 days for uncontrolled disease	34 (34.3)	20 (54.1)	54 (39.7)	0.049
Time from hospitalization to surgery within 30 days (median, IQR)	6 (3–9)	8 (6–15)	7 (5–11)	0.063
Death within 30 days	0 (0)	1 (2.7)	1 (0.7)	0.272

^*a*^ IS, immunosuppressors; WBC, white blood cells; PLT, platelets; Hb, haemoglobin; ESR, erythrocyte sedimentation rate; CRP, C-reactive protein.

**TABLE 2:** Characteristics of patients with CMV colitis

Overall 37 (27.4%)	
Endoscopic diagnosis	19/37 (51.4%)
Intraoperative diagnosis	18/37 (48.6%)
Method (IHC)	37/37 (100%)
Concomitant blood CMV-DNAemia	23 (62.2%)
CMV-DNA value (median, IQR; cp/mL)	1,008 (318–2,980)
Antiviral treatment	15 (40.5%)

### Comparison between patients with and without CMV colitis

A comparison between patients with and without CMV colitis was performed (Table 1). Patients with CMV colitis were on average older (60 vs 41 years, *P* = 0.004), with a higher median CCI (1 vs 0, *P* = 0.003). No differences regarding the management of UC, previous or during hospitalization, as well as clinical presentation at hospital admission were observed. However, a history of steroid-refractory disease was significantly associated with CMV colitis (86.5% vs 62.2%, *P* = 0.007). In addition, patients with CMV colitis had more frequent CMV-DNA blood detection (62.2% vs 10.1%, *P* < 0.001), with a higher median value (1,008 cp/mL vs 300 cp/mL, *P* = 0.013), and an increased rate of colectomy (54.1% vs 34.3%, *P* = 0.049). Kaplan-Meier analysis showed a trend toward a higher risk of colectomy in patients with CMV colitis (Log rank 3.26, *P* = 0.071).

### Risk factors associated to CMV colitis and 30-day colectomy

**Fig 1:**
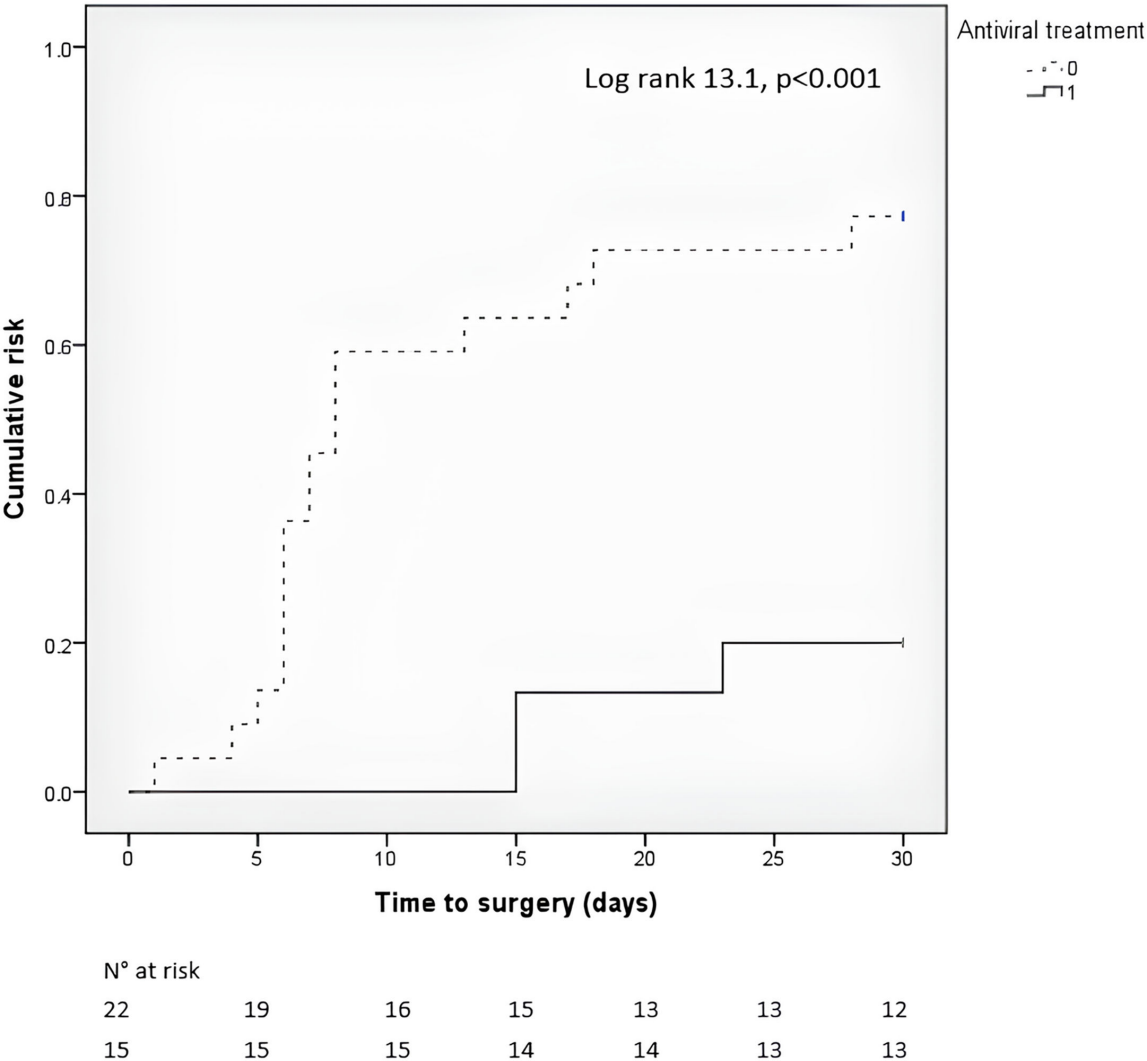
Kaplan-Meier between patients with CMV colitis treated or untreated with antivirals needing surgery (Log rank 13.1, *P* < 0.001).

A ROC analysis in order to evaluate the performance of CMV DNAemia was performed; a CMV-DNA cut-off ≥310 cp/mL showed a sensitivity of 85.7%, a specificity of 83.5%, a positive predictive value (PPV) of 65.7%, an negative predictive value (NPV) of 94.1%, and overall accuracy of 84.1% in predicting CMV colitis. At multivariable analysis for CMV colitis adjusted for age, CCI, steroid-refractory disease, and CMV-DNAemia, a blood threshold of 310 cp/mL (OR 25.04; 95% CI 6.53–95.98, *P* < 0.001), steroid-refractory disease (3.55; 95% CI 1.01–12.52, *P* = 0.049), and CCI (OR 1.64; 95% CI 1.15–2.32, *P* = 0.006) were independently associated with CMV colitis (Table 3). Finally, a multivariable analysis for 30-day surgery adjusted for main covariates was performed (Table 4): concomitant CMV colitis (OR 3.86; 95% CI 1.24–11.99, *P* = 0.019), steroid refractoriness (OR 3.82; 95% CI 1.52–9.60, *P* = 0.004), and age (OR 1.03; 95% CI 1.01–1.06, *P* = 0.009) were independently associated with increased risk of short-term colectomy. Excluding patients with CMV colitis diagnosed in surgical specimens, Kaplan-Meier curves demonstrated a significantly higher risk for colectomy in patients with concomitant CMV colitis untreated with antivirals (Log rank 13.1, *P* < 0.001; Fig. 1).

**TABLE 3:** Multivariable analysis for CMV colitis adjusted for age, CCI, steroid-refractory disease, and blood CMV-DNA

Variable	OR	95% CI	P
CMV-DNA ≥310 cp/mL	25.04	(6.53–95.98)	<0.001
Steroid-refractory disease	3.55	(1.01–12.52)	0.049
CCI	1.64	(1.15–2.32)	0.006

**TABLE 4:** Multivariable analysis for 30 days unplanned surgery adjusted for age, CCI, steroid-refractory disease, and CMV colitis

Variable	OR	95% CI	*P*
CMV colitis	3.86	(1.24–11.99)	0.019
Steroid-refractory disease	3.82	(1.52–9.60)	0.004
Age	1.03	(1.01–1.06)	0.009

## DISCUSSION

In this retrospective highly selected population of hospitalized patients for moderate-to-severe UC flare, CMV blood detection strongly predicts CMV colitis. As reported in previous studies, a history of steroid-refractory disease and a higher CCI were independently associated with CMV colitis. Patients with concomitant CMV colitis untreated with antivirals seem to have a significantly increased risk of short-term colectomy, underlying the need for prompt identification and risk stratification.

The impact of CMV in the management of patients with UC has been explored in several studies with discordant results. This issue is partially due to a heterogeneity of patient selection and definitions, along with micro- and macroscopic findings, reflecting inconsistent clinical outcomes. Given these premises, in order to minimize the risk of bias, we selected a well-defined population including only moderate-to-severe activity of hospitalized UC patients. Since positive IHC on bioptic specimens remains the cornerstone to CMV colitis diagnosis, we defined CMV colitis as positivity at IHC on colonic biopsies (5). With these definitions, CMV colitis prevalence in our cohort was 27.4%, in line with a recent literature review, attesting between 10% and 30% (11).

Generally, previous treatments for UC have been advocated as risk factors for CMV colitis development, although with substantial differences regarding specific drugs. In a retrospective cohort of 239 UC patients, anti-TNF-alpha agents were identified as an independent risk factor (12). In another cohort, other biological drugs than anti-TNF-alpha agents have been associated with CMV colitis (13). However, in this latter study, both UC and CD patients were enrolled, and patients were overall younger and treated with lower previous biological treatments compared with our cohort. Notably, a recent meta-analysis failed to demonstrate a correlation between previous infliximab administration and CMV reactivation (14), similar to our findings. Notably, different studies and meta-analysis demonstrated a noticeable relationship between steroid-refractory disease and CMV colitis (1, 15, 16). Accordingly, our study confirms that patients non-responders to high-dose steroid therapy are at high risk of concomitant CMV colitis.

Some authors assumed that a worse clinical presentation may be related to concomitant CMV colitis. In a retrospective cohort of 61 patients with UC, pancolitis was associated with CMV infection (17). However, in such a study, authors defined CMV infection as at least one positive test between serology, CMV-DNA detection on blood or biopsy. Conversely, in our study, we did not observe significant differences both in clinical presentation or endoscopic extension, but we considered only IHC as a reliable positive test.

As for laboratory findings at hospital admission, we observed a trend toward lower blood levels of albumin (3.3 g/dL vs 3.6 g/dl) in patients with CMV colitis compared to the control group. In this regard, hypoalbuminemia, a laboratory marker of possible malnutrition, has previously been associated with CMV colitis onset (18).

Several studies highlighted the impact of CMV colitis on poorer outcomes (17–19). In a multicenter study, colectomy and disease flare-up rates were significantly higher in CMV infected compared to uninfected patients (20). To notice, some authors reported that a higher CMV IHC-positive cells on colonic biopsy is indicative of a greater colectomy risk, suggesting that the higher the colonic viral load, the higher the risk of colectomy, supporting the benefit of antiviral therapy (21).

One of the most important findings of our study is that even a low CMV DNA load in blood seems to predict CMV colitis. Several studies explored this issue without a definitive response. When compared to tissue-based testing, CMV-DNA detection in blood had a range of sensitivity between 44% and 60%, with a specificity up to 88% (22, 23). Furthermore, in this selected population, a specific cut-off able to distinguish between a non-clinically relevant reactivation and CMV colitis has not been addressed. In our cohort, a cut-off of 310 cp/mL showed a sensitivity of 86% and a specificity of 84%. In addition, such a threshold had an NPV of 94%, potentially able to rule-out a suspected CMV colitis if active replication on blood is not detected. These findings are quite unexpected, in particular, if we compare cut-offs typically applied in transplant recipients deserving of antiviral treatment.

When concomitant CMV colitis is diagnosed in moderate-to-severe UC patients, antiviral treatment could probably be seen as a time-dependent variable able to reduce the risk of colectomy. Furthermore, it has been reported that untreated patients with CMV colitis may have colitis flare-ups after the index admission (24). A recent meta-analysis found that among patients with a steroid-refractory disease, antiviral therapy resulted in a significantly lower risk of colectomy (OR 0.20; 95% CI 0.08–0.49) (25). Accordingly, in our study, patients promptly treated with antiviral therapy had a significant reduction in short-term colectomy (Log-rank 13.1, *P* < 0.001). Considering that patients with concomitant CMV colitis had an intrinsic major risk of surgery, a prompt identification of these patients could positively impact their short- and long-term outcome, and CMV-DNA blood testing should be part of a detailed diagnostic work-up.

Limitations should be addressed. First, our results may be influenced by the monocentric observational nature of the study. However, our sample size is consistent and well-characterized. In addition, the study period is relatively short, allowing for a standardized management of hospitalized patients with UC flare which was adopted throughout the study period. Unfortunately, we could not assess the value of CMV-PCR on colonic biopsies, which it may have added value at our study. Nevertheless, such method is not standardized, and the clinical significance of a positive PCR without other histological signs of infection remains unclear; thus, we decided to include only patients with histological signs of tissue invasion along with positive IHC. Further studies addressing the role of tPCR should be conducted.

In conclusion, CMV-DNAemia, along with a history of steroid-refractory disease, is significantly associated with concomitant CMV colitis among patients hospitalized for moderate-to-severe UC flare. Since the risk of colectomy is high among patients with concomitant CMV infection, and still higher in untreated patients, a pre-emptive administration of antivirals, awaiting for colonic biopsies results, could be justified. Further prospective studies, including CMV-DNA blood testing for diagnostic work-up in this specific setting, are strongly needed.

### Key messages

- **What is already known?** CMV colitis worsens the prognosis of patients with active ulcerative colitis, increasing the risk of poor outcomes as hospitalization and colectomy.
- **What is new here?** CMV blood reactivation is independently associated with CMV colitis in patients with moderate-to-severe UC and can be detected earlier than the gold standard for CMV colitis (immunohistochemestry and tissue PCR on biopsies).
- **How can this study help patient care?** Early identification of markers associated with CMV colitis in patients with active UC can lead and antiviral treatment and reduce the risk of colectomy.
